# Calcium-sensing receptor promotes calcium oxalate crystal adhesion and renal injury in Wistar rats by promoting ROS production and subsequent regulation of PS ectropion, OPN, KIM-1, and ERK expression

**DOI:** 10.1080/0886022X.2021.1881554

**Published:** 2021-03-08

**Authors:** Xiaoran Li, Siyu Chen, Demei Feng, Yuqiang Fu, Huang Wu, Jianzhong Lu, Junsheng Bao

**Affiliations:** Department of Urology, Institute of Urology, Gansu Nephro-Urological Clinical Center, Key Laboratory of Urological Diseases in Gansu Province, Lanzhou University Second Hospital, Lanzhou, People’s Republic of China

**Keywords:** CaSR, PS ectropion, crystal adhesion, ROS, calcium oxalate

## Abstract

**Objectives:**

To explore the mechanism of calcium-sensing receptors (CaSRs) during the development of nephrolithiasis.

**Materials and methods:**

Wistar rats were treated with ethylene glycol to induce calcium oxalate crystallization, and gadolinium chloride (GdCl_3_, an agonist of CaSR) and NPS 2390 (an antagonist of CaSR) were added. Oxidative stress (OS) and calcium oxalate crystals in the kidney were observed. CaSR expression and the expression of extracellular signal-regulated protein kinase (ERK), OPN, and KIM-1 were determined by western blotting. In addition, renal tubular epithelial cells were isolated from the kidney to observe phosphatidylserine (PS) ectropion using flow cytometric analysis. Various biochemical parameters were assessed in serum and urine at the end of the experiment.

**Results:**

Calcium oxalate increased OS, crystal adhesion, PS ectropion, and the expression of CaSR and ERK, OPN, and KIM-1 *in vivo*. In addition, lower levels of urine citrate as well as increased serum creatinine and urea levels were observed after treatment with calcium oxalate (*p* < .05). Compared with calcium oxalate treatment alone, the above deleterious changes were further significantly confirmed by GdCl_3_ but were reversed by NPS-2390. However, urine calcium excretion was decreased after ethylene glycol treatment but was significantly reduced by NPS 2390 and increased by GdCl_3_ (*p* < .05).

**Conclusions:**

The results suggest that CaSR might play significant roles in the induction of nephrolithiasis in rats by regulating reactive oxygen species (ROS) and PS ectropion and the composition of urine, OPN, KIM-1, and ERK expression.

## Introduction

Numerous studies have confirmed that the incidence and prevalence of nephrolithiasis are increasing remarkably [1]. As a complex multifactorial disease, nephrolithiasis results from interactions between genetic factors and the environment. Approximately, 70% of human nephrolithiasis is primarily composed of calcium oxalate [2]. Studies have shown that a high concentration of oxalate could lead to renal injury and contribute considerably to the deposition and progression of calcium oxalate crystals [3]. A large amount of evidence suggests that increased calcium oxalate crystal adherence to and aggregation in renal tubular cells are related to the overproduction of reactive oxygen species (ROS) [4]. ROS result in serious renal tissue injuries, such as lipid peroxidation of the cellular membranes, presumably through OS [5].

CaSR, a dimeric G protein-coupled receptor, plays an important role in calcium homeostasis. The CaSR regulates parathyroid hormone release and calcium reabsorption in the parathyroid glands and kidneys at the highest levels [6,7]. CaSR responds to numerous ligands, including extracellular Ca^2+^, trivalent cations, antibiotics, amino acids, and other external stimuli, such as ionic strength and pH [8]. The polymorphism of the CaSR gene Arg990Gly was related to hypercalciuria and nephrolithiasis [9]. Studies indicate that *CaSR* is a candidate gene to explain the individual predisposition to calcium stones [10]. A published study suggested that CaSR was part of a common pathway in nephrolithiasis formation [11]. In our previous study, we found that CaSR is present in renal tubular epithelial cells and rat kidneys. Notably, CaSR upregulation might activate intracellular signaling pathways, potentially leading to damage to the kidney and ultimately stone retention [5].

Growing studies have indicated that calcium oxalate crystal deposition in the kidneys is associated with renal epithelial injury since Randall emphasized the importance of renal damage in stone formation [12]. Many studies have shown that inflammation and OS are significant pathologies for nephrolithiasis formation [13]. Clinical studies have also reported that patients with kidney stones are under oxidative stress (OS) [14]. Excessive ROS produced by renal tubular epithelial cells are due to OS under the condition of high oxalic acid stimulation, resulting in epithelial cell damage, inflammation, and ultimately apoptosis [15]. Cell damage induced by ROS is the initial mechanism of crystal nucleation, attachment, and retention [13,16].

Phosphatidylcholines, choline phospholipids, and sphingomyelins are concentrated in the exoplasmic (outer) leaflet, while aminophospholipids, PSs, and most phosphatidylethanolamines are predominantly located on the cytoplasmic (inner) leaflet [17]. Membrane proteins established and maintained asymmetrical distribution of phospholipids. Membrane proteins transport phospholipids between the exoplasmic (outer) leaflets and cytoplasmic (inner) leaflets.

One study showed that oxalic acid-induced ROS production could lead to PS externalization on the renal epithelial cell membrane, while negatively charged PS exposed to the cell surface might act as anion molecules mediating crystal adhesion [18]. Treatment with apocynin, an ROS inhibitor, significantly reduced crystal deposition, tubule injury and dilation, renal expression of KIM-1, OPN, and ED-1, and excretion of KIM-1, OPN, MCP-1, and H_2_O_2_ in renal calculus rats [16]. In addition, the production of ROS activates the extracellular signal-regulated protein kinase (ERK) signaling pathway, which determines the cellular fate of different cell types [19,20]. In mammalian cells, ERK kinases play an important role in regulating fundamental intracellular processes, such as cell differentiation, proliferation, and death [21].

The present study was designed to examine the possible effects of CaSR in a calcium oxalate nephrolithic model and to provide direct pharmacological evidence to determine whether CaSR is involved in this process.

## Materials and methods

### Materials

Quinoxaline-2-carboxylic acid adamantan-1-ylamide (NPS2390, CAS number 226878-01-9) was obtained from Tocris Bioscience (Minneapolis, MN), GdCl_3_ (product number 43977-0) was obtained from Sigma-Aldrich (St. Louis, MO). Anti-OPN (ab36125), anti-phospho-ERK (ab21396), and rabbit polyclonal antibodies against KIM-1 were obtained from Abcam (Abcam Inc., Boston, MA). Antibodies against the calcium-sensing receptor (CaSR, sc33821, rabbit) and glyceraldehyde 3-phosphate dehydrogenase (GAPDH; sc32233, mouse) were purchased from Santa Cruz Biotechnology, Inc. (Dallas, TX). Fluorescein isothiocyanate (FITC)-labeled annexin V (annexin V-FITC) was obtained from BD Pharmingen (San Diego, CA). Ethylenediaminetetraacetic acid (EDTA) was obtained from Sigma-Aldrich Co., Ltd. (St. Louis, MO). An ECL chemiluminescence kit (WBKLS0500) was obtained from Millipore Corporation (Billerica, MA).

### Experimental animals and animal protocol

All of the rat experiments were performed under protocols approved by the Institutional Animal Care and Use Committee of Lanzhou University. Male Wistar rats, aged 6–8 weeks, were purchased from Lanzhou University Medical Laboratory Animal Center (Lanzhou, China). Rats were housed in polypropylene cages and had access to food and water. The rats were divided into four equal groups. Group A: the intact control group was given tap water as their drinking water and fed a standard diet. Group B: the rats were exposed to 1% EG in their drinking water for 4 weeks to induce nephrolithiasis. Group C: animals were exposed to 1% EG in their drinking water, and an 8.67 mg/kg dose of GdCl_3_ was administered daily by injection for 4 weeks. Group D: animals were exposed to 1% EG in their drinking water, and a 0.20 g/kg dose of NPS-2390 was administered daily by injection for 4 weeks [22].

### Analysis of urine samples

On day 28, the rats were placed in metabolic cages for 24-h urine sample collection before sacrifice. The urine sample was either stored at −80 °C or analyzed immediately. Urinary pH, calcium, uric acid, and magnesium levels were determined by an automated analyzer (model 705, SRL, Tokyo, Japan). Oxalate in urine samples was analyzed by direct precipitation followed by titration. A Citric Acid Enzyme Bioanalysis Kit (Megazyme, Wicklow, Ireland) was used to measure citrate levels.

### Kidney and blood sample collection and analysis

Approximately, 3 mL of blood samples was collected from the jugular vein into heparinized syringes and was immediately placed on ice. Whole samples were transferred to serum separator tubes and centrifuged at 3000 rpm for 10 min to isolate the plasma, which was then subjected to SOD, MDA, and renal function analysis (Hitachi7600, Tokyo, Japan).

The animals were sacrificed under anesthesia; both kidneys were removed and washed with cold 0.9% NaCl. The left kidney was cut in half; one half was stored for tissue microbiological studies, primary culture and identification of Wistar rat renal tubular epithelial cells, and the other half was fixed in 10% formalin, embedded in paraffin, cut into 5-µm-thick sections, and stained for histopathology (hematoxylin and eosin). The right kidney was cut in half; one half was minced with scissors and then homogenized in 0.9% NaCl using a glass homogenizer. The homogenate was centrifuged at 2000 rpm for 10 min in a refrigerated centrifuge to remove cell debris. Tissue MDA and SOD levels were determined in the supernatant using commercially available kits (Jiancheng Bioengineering, Nanjing, China). The other half of the kidney was immediately placed in liquid nitrogen and stored at −80 °C for western blotting analyses.

### Renal crystal deposits and pathological examination

As mentioned earlier, crystal deposition in the kidney was assessed using the following score: no deposition = 0; crystal deposition at the tip of the nipple = 1 point; crystal deposition at the cortical medullary junction = 2 points; and crystal deposition in the cortex = 3 points. If crystal deposition is observed at multiple sites, the points are combined to provide a total score for each pathological section. Pathological changes were semiquantified according to the damage area: 0–3, 0 = invisible lesions; 1 = tubulointerstitial inflammatory infiltration in the lesion area/20%, slight tubule dilatation; 2 = tubulointerstitial inflammatory infiltration in the lesion area/40%, tubules significantly expanded; 3 = tubulointerstitial inflammatory infiltration in the lesion area/40%, tubules severely dilated. For further observation of the crystals, transparent tissue portions of the crystals (without HE staining) were also observed under a laser-scanning confocal microscope (LSM 510, Karl-Zeiss, Jena, Germany).

### Microbiological studies

On day 28, urine from separate metabolic cages was also assessed for microbial variation. Half of the left kidney was homogenized separately in sterile saline (5 mL). The homogenate was diluted (10^−1^, 10^−3^, and 10^−5^) before the tissue homogenates were cultured on plates, and the bacterial count was performed after the dilution factor was corrected. Standard microbiology techniques were carried out. Urinary infection was defined as more than 10^5^ colony-forming units per mL (cfu/mL) in urine. Renal tissue ≥10^5^ cfu/g suggests pyelonephritis.

### Primary culture and identification of Wistar rat renal tubular epithelial cells for PS detection

*Primary culture*: Half of the left kidney was stored in a fume hood, placed on ice-cold PBS to remove the renal capsule, and then placed on an 80 mesh stainless steel mesh screen. Crush and fully grind, then rinse thoroughly with serum-free F12. The filtrate was filtered through a 160-mesh stainless steel mesh and thoroughly washed with F12, and the renal tubule segments on the net were collected. After fully aspirating, transfer to a centrifuge tube. Centrifuge at 1000 r/min for 5 min. Discard the supernatant after centrifugation, add 0.05% trypsin + EDTA 2 mL, and digest at 37 °C. Shake once every 3 min. After 20 min, an equal volume of F12 culture medium containing 10% FBS was added to terminate the digestion. Centrifuge at 1000 r/min for 5 min. Discard the supernatant, add F12 containing 10% serum, mix thoroughly, transfer to a petri dish for culture (37 °C, 5% CO_2_). Seventy-two hours for the first fluid change, after every two-day fluid change.

*Identification of Wistar rat renal tubular epithelial cells: morphology*: Inverted microscopy revealed that the cells were larger in volume, appeared as multilateral cobblestones, had strong transparency and refractive sex and were closely connected. *Immunocytochemistry*: After passage for one generation, renal tubular epithelial cells were seeded in coverslips in a 24-well cell culture plate, and the cover glass was removed when the cells were approximately full. Rinse twice in PBS, fix in 4% cold paraformaldehyde for 30 min. After two PBS rinses, add 3% H_2_O_2_ deionized water and incubate for 10 min to prevent peroxidase, cytokeratin 18 primary antibody (1:400) at 4 °C overnight (the negative control uses PBS instead of the primary antibody). The secondary antibody (ready-to-use) was incubated at 37 °C. Incubate for 1.5 h, then develop DAB color, rinse with tap water, hematoxylin counterstain, and remove water, transparent, coverslip. The results were observed under an ordinary light microscope. Detection of renal tubular epithelial cells by immunocytochemical staining: heterosexually expressed cytokeratin 18.

### PS ectropion by flow cytometric analysis

Annexin V-FITC was used as a marker for the detection of phosphatidylserine (PS). Negative controls for annexin V binding were stained with annexin V-FITC in the presence of 2.5 mM EDTA instead of 2.5 mM CaCl_2_. Samples were analyzed though a FACS Calibur flow cytometer (Becton Dickinson, Franklin Lakes, NJ) equipped with an argon-ion laser emitting at 488 nm. The fluorescence channels and light scatters were set on a log scale. Data from 5000 events were collected and analyzed using Cell Quest Pro software.

### Western blotting analysis

The kidney tissues were homogenized in 1 mL lysis buffer mixed with 10 μL phenylmethanesulfonylfluoride (100 mM) and NaF (100 mM) for 10 s on ice. After centrifugation (12,000×*g* for 5 min) at 4 °C, supernatants were collected. The protein concentration was measured using a Bradford Protein Assay Kit (Bio-Rad, Hercules, CA). Then, samples were heated at 95 °C in 2.5% mercaptoethanol and Laemmli sample buffer (Bio-Rad, Hercules, CA), and a total of 25 µg of protein was loaded per lane onto 4–20% Mini-PROTEAN TGX Precast gels (Bio-Rad, Hercules, CA). Following separation at 130 V, the proteins were transferred onto trans-blot turbo transfer pack membranes (Bio-Rad, Hercules, CA). PVDF blocking reagent was used to block the membranes for 1 h at room temperature. After washing with Tris-buffered saline with Tween 20 (TBST, Takara Bio, Kusatsu, Japan) three times, the membranes were incubated with antigen-specific antibodies at 4 °C overnight. Then, the membranes were incubated with a horseradish peroxidase (HRP)-conjugated secondary antibody for 1 h at room temperature. Finally, the immunoreactive bands were visualized with enhanced chemiluminescence western blotting reagent.

### Statistical analysis

The data are presented as the mean ± standard error. Significance was evaluated by Student’s *t*-test for comparisons between two groups or one-way ANOVA for comparisons of three or more groups. A *p* value less than .05 was considered significant.

## Results

### Biochemical changes in urine and serum

As shown in Table 1, urinary calcium concentration showed a reduction in group B compared with group A, and urinary calcium was significantly higher in group C than in group A and group B (*p* < .05). However, urinary calcium was significantly reduced in group D compared with group A and group B (*p* < .05).

**Table 1.** Urine biochemical parameters and serum parameters of SD rats.

Parameter (unit)	Group A	Group B	Group C	Group D
*Urine parameters*				
pH	6.45 ± 1.32	6.51 ± 1.19	6.82 ± 0.98	6.13 ± 1.09
Calcium (mmol/L)	2.28 ± 0.53	2.07 ± 0.35	5.81 ± 0.69^@,#^	1.57 ± 1.05^@,#^
Oxalate (μmol/L)	125.3 ± 18.7	868.9 ± 87.4^@^	483.4 ± 68.7^@,#^	1154.8 ± 138.1^@,#^
Magnesium (μmol/L)	6.42 ± 1.48	6.24 ± 1.55	6.01 ± 1.02	6.37 ± 1.10
Citrate (mmol/L)	0.73 ± 0.12	0.70 ± 0.31	0.46 ± 0.07^@,#^	1.65 ± 0.17^@,#^
Uric acid (mmol/L)	2.88 ± 0.31	2.49 ± 0.39	2.40 ± 0.44	2.49 ± 0.53
*Serum values*				
Creatinine (mmol/L)	34.33 ± 6.07	80.76 ± 18.62^@^	106.31 ± 9.2^@,#^	50.68 ± 8.71^@,#^
Urea (μmol/L)	8.36 ± 1.58	24.89 ± 6.79^@^	40.78 ± 5.22^@,#^	15.58 ± 3.25^@,#^

^@^*p*<.05 compared to group A, ^#^*p*<.05 compared to group B, *n* = 10.

Hyperoxaluria was evident in group B on the 28th day and was subsequently reduced by GdCl_3_ administration in group C but was increased in group D compared with group B (*p* < .05). The urinary citrate concentration was also lower in group B than in group A, and after treatment with GdCl_3_ in group C, the urinary citrate concentration decreased significantly, but NPS2390 treatment group D showed a significant increase in the urinary citrate concentration. Group B had three times higher values of blood urea and two times higher values of serum creatinine than group A, and treatment with NPS2390 nearly reduced the two indices to normal concentrations. However, treatment with GdCl_3_ further impaired renal function compared to group B (*p* < .05).

### Calcium oxalate crystal deposition and histological examination

**Figure 1.**
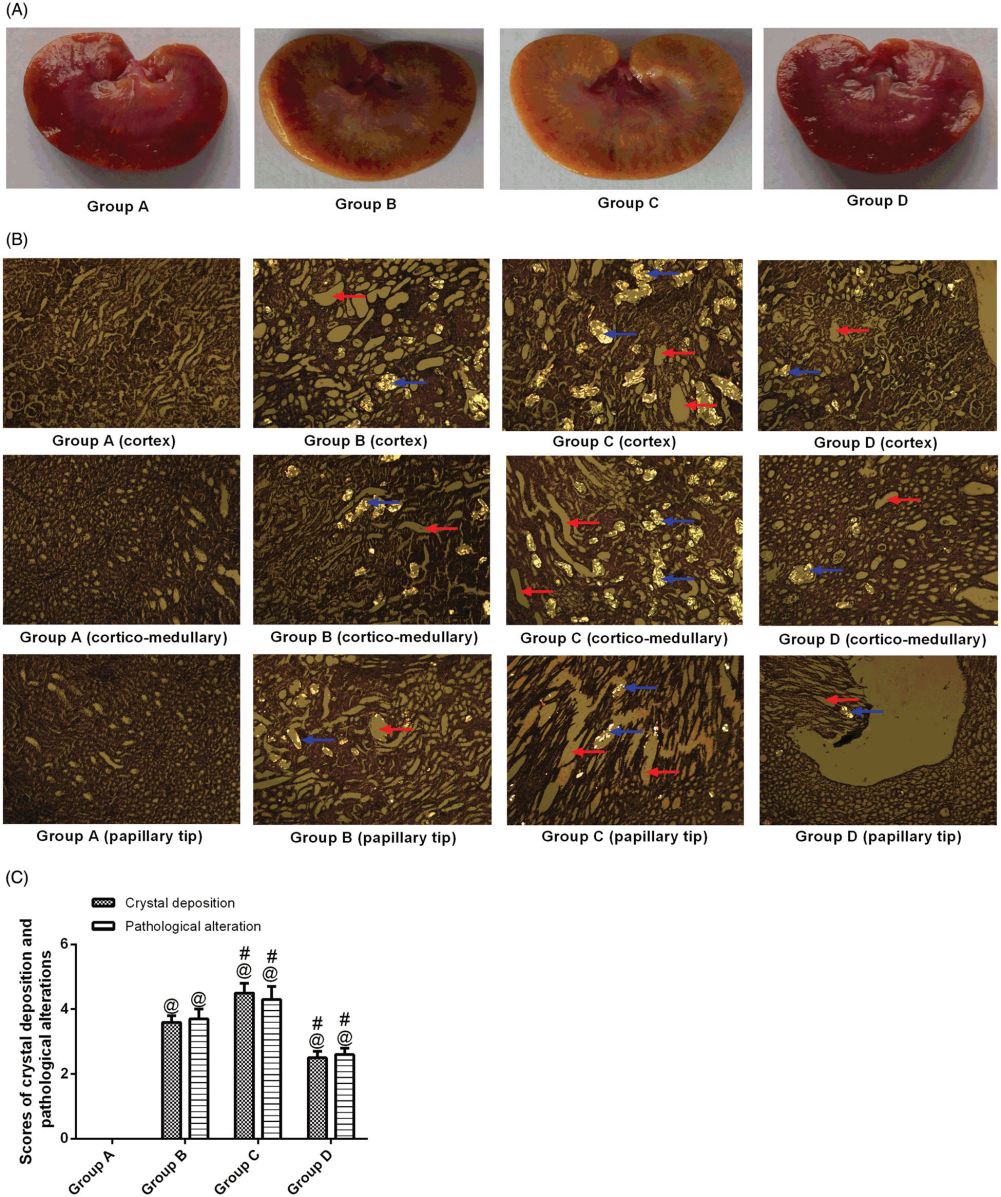
Histopathology and crystal deposition in the rat kidney. (A) Gross anatomy of the kidney (left to right: groups A–D). (B) Micrograph of HE-stained renal 7tissue under a polarizing microscope. (C) Pathological alterations and calcium oxalate deposition scores. Blue arrows indicate renal crystals; red arrows indicate dilatation of the renal tubules. The data are presented as the mean ± SD. ^@^*p*<.05 compared with group A; ^#^*p*<.05 compared with group B. (Color figure online)

HE staining of renal tissues from group A revealed regular histology with normal tubules with a single epithelial lining along the margin and did not reveal any crystal deposition (Figures 3(A) and 1(B)). As shown in Figure 1(B), ethylene glycol-treated rats had significant crystal deposition (blue arrows) in all three major parts of kidney sections. The crystal deposition scores were significantly higher in group C than in group B (Figure 1(C)) (*p* < .05). There were fewer crystal deposits in group D than in group B (Figure 1(C)) (*p* < .05). Figure 1(A) also shows gross pathological changes, such as renal swelling in group B, but were not found in the kidneys of the animals in group D, indicating that inducing stones with 1.0% ethylene glycol throughout the entire 4-week experimental period did lead to gross renal alterations. The gross alterations observed in the kidneys of group C were similar to those of group B.

**Figure 2.**
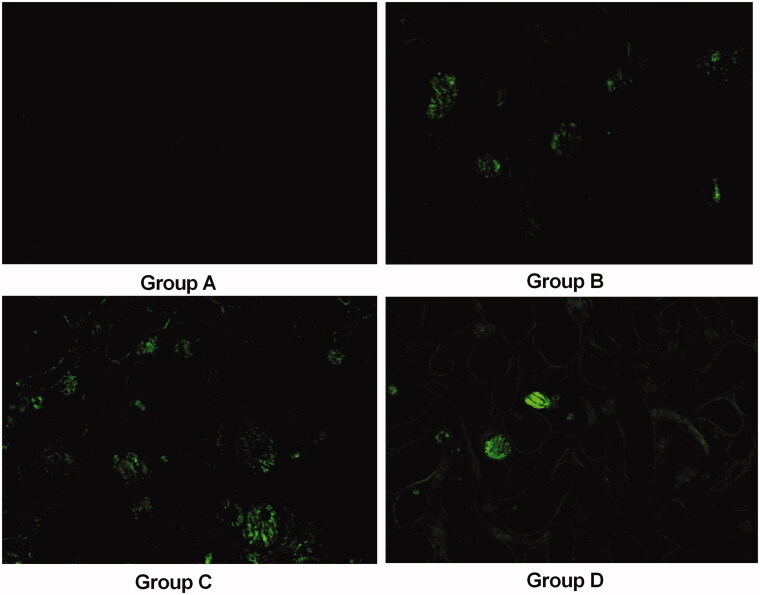
Representative confocal pictures of crystals in transparent renal sections (without HE staining) in groups A–D (100×).

The renal tissue from the animals in group B was severely damaged (Figure 1(B)). Indeed, degeneration of the epithelial lining, focal mild degeneration of tubules of the medulla, enlarged renal tubules (red arrows) with amorphous deposits in the lumen and multifocal minimal intertubular lymphocytic infiltration (Figure 1(B)) were observed. Improvements were noted in the renal tissues from group D; the pathological alterations in the animals in group D revealed slight lymphocytic infiltration and mild tubules (Figure 1(C)). In contrast, group C had a higher pathological score than group B. The crystals in transparent tissue sections of groups A–D (Figure 2(A-D)) were also observed with a laser-scanning confocal microscope, which showed that the crystals exhibit spontaneous fluorescence.

### Flow cytometric detection of PS exposure

**Figure 3.**
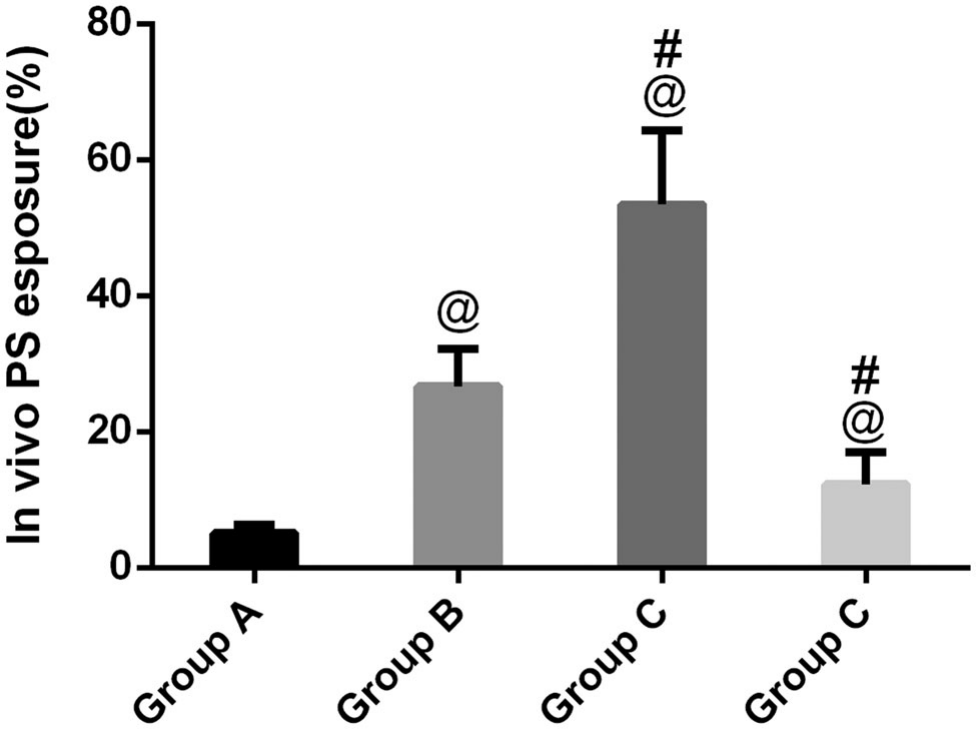
Flow cytometric detection of PS exposure. A regular distribution of PS is shown after calcium oxalate treatment in group B. No PS exposure was observed in group A. In group C, calcium oxalate-treated rats induced by GdCl_3_, PS exposure of such rats significantly increased in renal tubular epithelial cells, and in group D, PS exposure decreased compared with group B. ^@^*p*<.05 compared with group A; ^#^*p*<.05 compared with group B.

Phosphatidylserine exposure has been suggested as an important event in kidney stones, which frequently concurs with morphological alterations from cells into tissue. In Figure 3, as expected, annexin-positive cells could be observed in group B, indicating that calcium oxalate-induced PS exposure is the regulated event occurring on the outer membrane of renal tubular epithelial cells. In contrast, with flow cytometry, compared to group B, GdCl_3_ significantly increased PS exposure (*p* < .05). Furthermore, after treatment with NPS-2390, the remaining renal tubular epithelial cells in group B did not express much PS exposure, which was in clear contrast with group C.

### Microbiological studies

**Figure 4.**
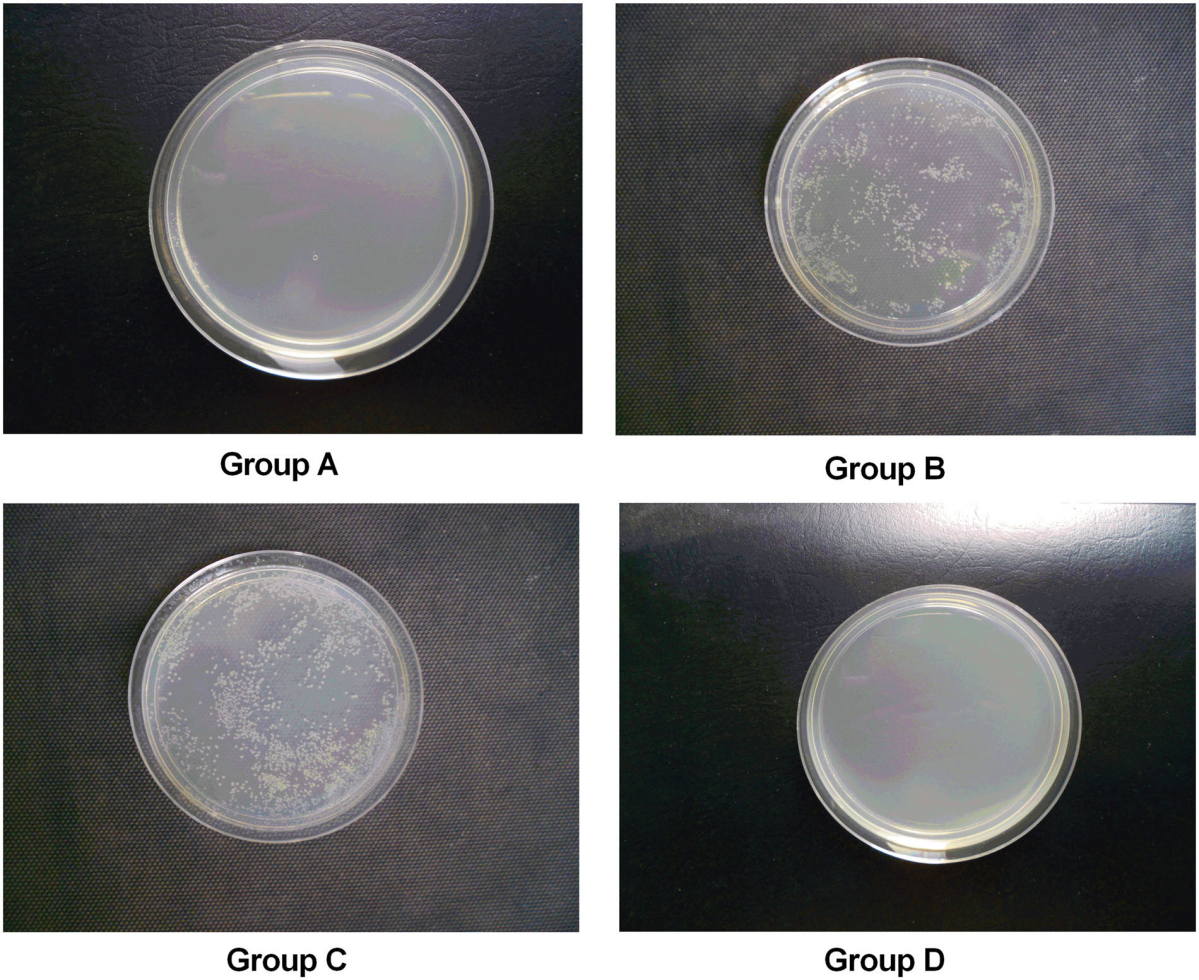
Bacterial urine cultures. (A) group A; (B) group B; (C) group C; and (D) group D.

Damage to the kidney leads to crystal deposition, and bacterial attack might develop, leading to full crystal deposition in the kidney. As shown in Figure 4, no rats from group A (0%) showed a urinary tract infection (UTI), whereas the UTI rate in group B was 100% (10/10). After treatment with NPS-2390, the UTI rate was 20% (two of 10) in group D (*p* < .05 compared with group B), indicating that NPS-2390 could protect the urinary tract from bacterial infection. However, the UTI rates were 80% (eight of 10) in group C (*p*>.05 compared with group B). The incidence of pyelonephritis (0%, 100%, 80%, and 20%) in groups A, B, C, and D was consistent with the UTI rate.

### Changes in SOD and MDA levels in Wistar rats in the four groups

**Figure 5.**
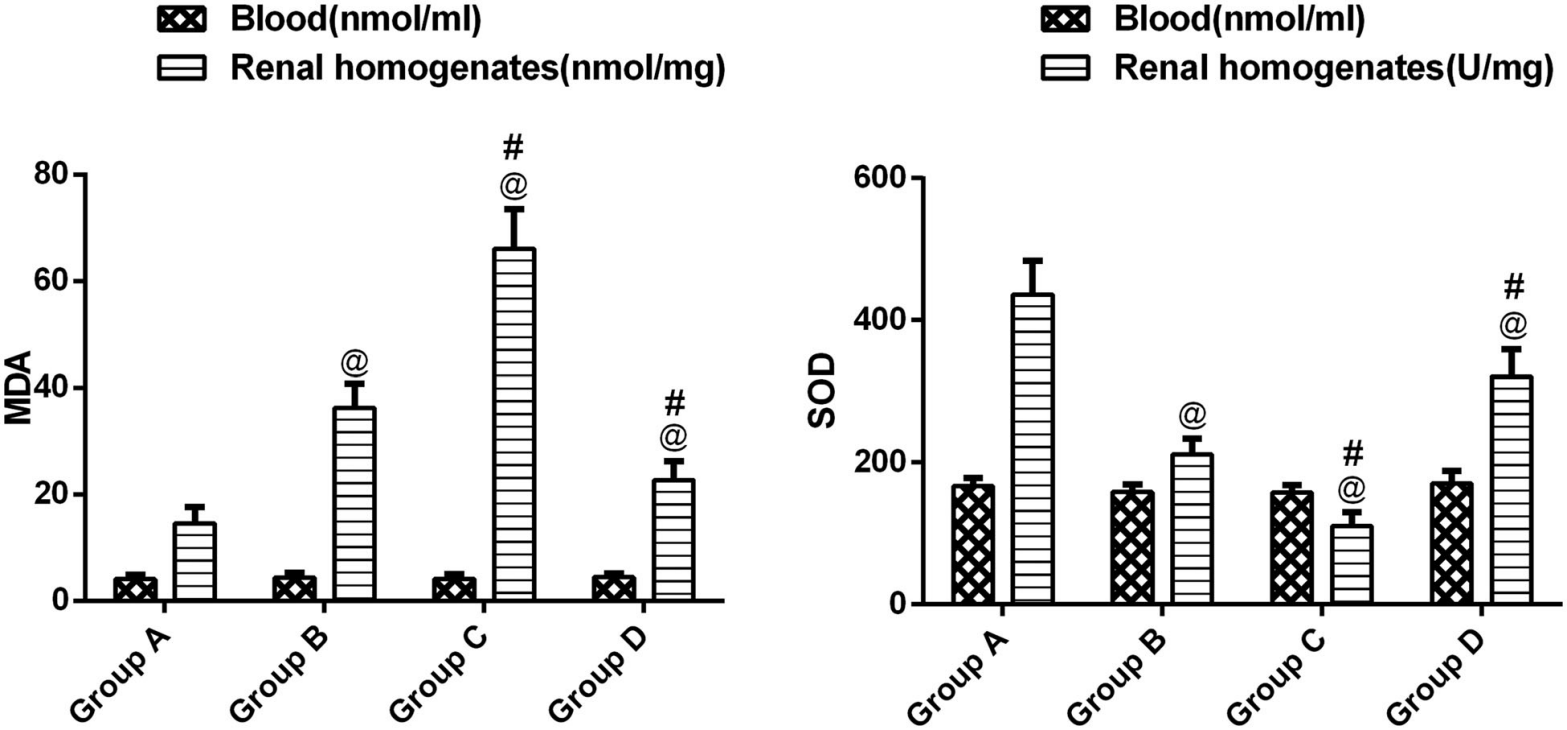
Levels of MDA and SOD activity in blood and kidney in different groups. ^@^*p*<.05 compared with group A; ^#^*p*<.05 compared with group B.

Elevated MDA levels were detected in group B, while SOD levels were in the lower range compared with untreated rats (*p* < .05) (Figure 5). The evaluation of group C revealed an elevated level of MDA and decreased SOD level, which indicated a marked increase in ROS.

However, when CaSR expression was decreased by NPS-2390, SOD activity was restored, and MDA and levels were reduced compared with group B (*p* < .05).

### Western blot analysis for protein in kidney

**Figure 6.**
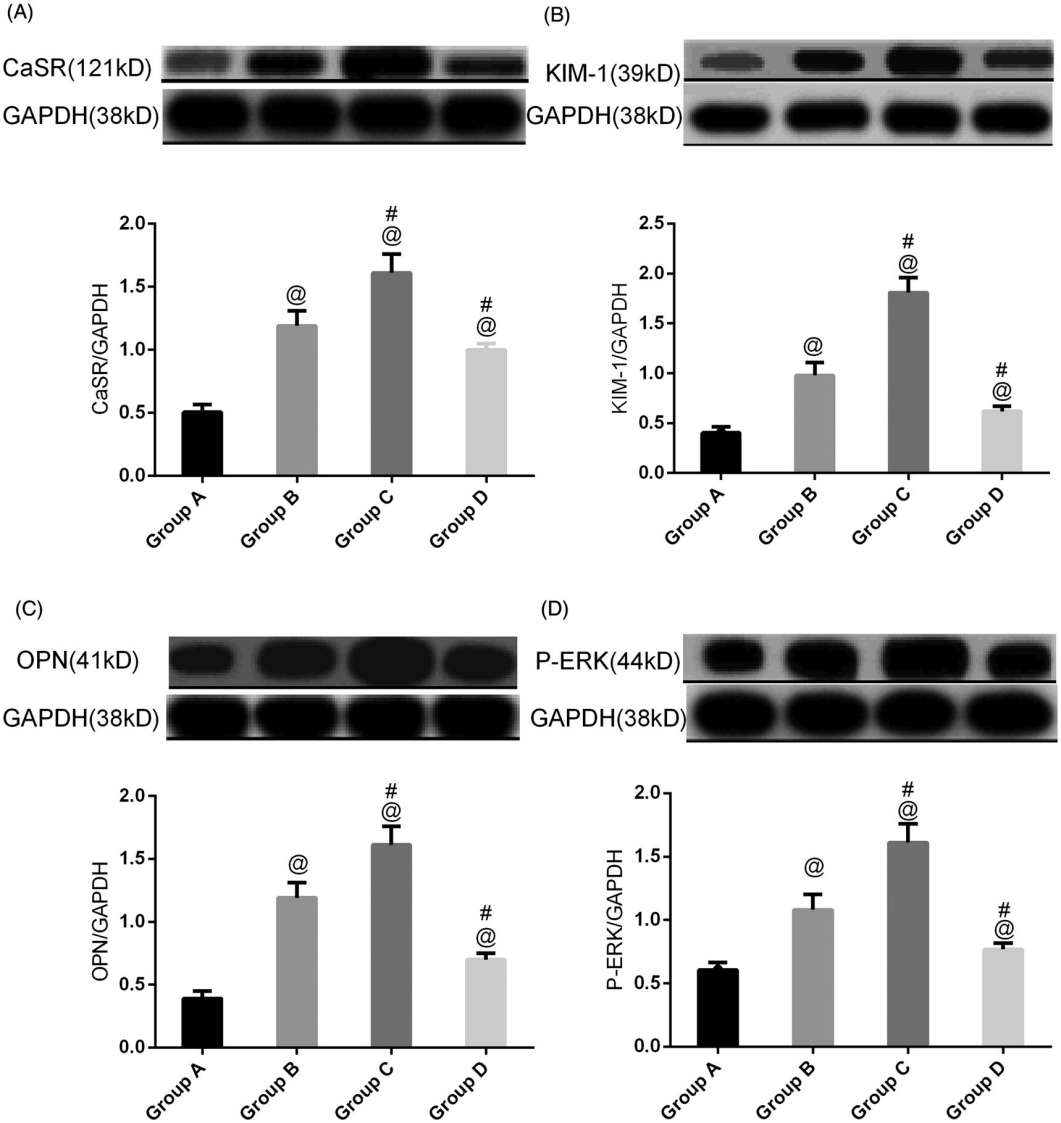
CaSR expression, ERK, OPN, and KIM-1 in rat kidneys in different groups. All the data are expressed as the mean ± SEM (*n* = 5). ^@^*p*<.05 compared with group A; ^#^*p*<.05 compared with group B.

To clarify the protein involved in nephrolithiasis formation in our rat model, western blot analysis was carried out on total protein extracted from rat kidneys. First, more than twofold changes in CaSR expression in group B compared with group A were detected (Figure 6(A)). Upregulated CaSR in group C was detected in group C compared to group B. CaSR expression in group D was lower than that in group B (*p* < .05).

To further validate the stone-related protein expression changes, a semiquantitative western blot of ERK, KIM-1, and OPN expression was carried out with the four groups of animals.

Ethylene glycol treatment significantly (*p* < .05) increased ERK, KIM-1, and OPN expression in group B compared to normal animals. Treatment with NPS2390 produced a significant (*p* < .05) reduction in ERK, KIM-1, and OPN levels compared to group B. However, the elevated levels of ERK, KIM-1, and OPN were significantly further increased by treatment with GdCl_3_ in group C (*p* < .05) (Figure 6(B–D)).

## Discussion

CaSR has been found to be expressed in all key tissues involved in extracellular Ca^2+^ homeostasis, including parathyroid cells, thyroidal calcitonin-secreting C-cells [23], kidney [24], and intestine [25].

There is growing evidence that CaSR may play a role in increasing the propensity for kidney stones. Our previous studies have shown that CaSR is present in HK-2 cells and rat kidneys, and it is worth noting that CaSR upregulation may increase intracellular MAPK signaling, which may lead to crystal retention and ultimately damage renal function [5]. Studies have shown that renal epithelial cells respond to calcium oxalate crystals and increase the production of macromolecular proteins, such p38, such as osteopontin (OPN), hyaluronic acid, a-1-microglobulin, Tamm-Horsfall protein, and prothrombin fragment-1, which in turn adjust the crystal deposition process [26,27].

In the present study, we used gadolinium chloride (GdCl_3_, agonist of CaSR) and NPS 2390 (antagonists of CaSR) to explore the effects of CaSR on nephrolithiasis in rats. The expression of CaSR was significantly increased after ethylene glycol treatment for 28 days and was further significantly increased when cotreatment with the CaSR agonist GdCl_3_ but was attenuated by NPS-2390. Except for many cations (e.g., Gd^3+^ and Mg^2+^), polyamines, and some antibiotics can activate CaSR [28], in our present study, we found that calcium oxalate crystals could activate CaSR expression in rat kidneys.

In addition, the levels of OS, macromolecular protein, PS ectropion, UTI, renal dysfunction, and massive calcium oxalate deposition were all significantly increased under ethylene glycol treatment for 28 days. Furthermore, these changes could be weakened or enhanced by either a CaSR agonist or its antagonist, suggesting that these indicators could be regulated by CaSR.

Cao et al. found that high oxalate promoted PS redistribution and crystal adhesion increase in renal epithelial cells and supported the idea that oxalate toxicity may lead to nephrolithiasis by altering the properties of renal epithelial cell membranes [29]. PS exposure to the surface of renal papillary epithelial cells mediates the attachment of stone crystals to renal papillary tubular epithelial cells, which may be the initiation event of renal calculi [30]. The percentage of Annexin V-FITC-positive cells was greater in group B than in the control group (*p* < .05). This finding indicates that PS exposure on the renal cell membrane surface increased under the action of calcium oxalate crystals. GdCl_3_ further enhanced PS exposure, while NPS-2390 decreased it (*p* < .05), which suggests that CaSR helps to enhance PS exposure.

Urinary tract infection is reported to be associated with renal calculi, especially struvite, which is the result of being infected by urea-splitting bacteria, such as *Proteus mirabilis* [31,32]. However, one study has indicated that not only struvite but also calcium oxalate nephrolithiasis are associated with UTI; in addition, *E. coli*, not *P. mirabilis*, is the most common bacterium found in the urine and stone matrix of stone patients [33]. However, it remains to be clarified whether *E. coli* plays a pathogenic role in the formation of nephrolithiasis or whether it is confined to the stone matrix. Interestingly, previous studies have indicated that *E. coli* could promote the growth and aggregation of calcium oxalate crystals [34,35], both of which are significant processes in stone formation. In the present study, calcium oxalate-induced CaSR significantly aggravated UTIs, which may promote crystal formation.

In this study, we evaluated the effects of CaSR on stone-related proteins in rats exposed to ethylene glycol using agonist (GdCl_3_) and antagonist (NPS 2390) CaSR. We found increases in the levels of OPN, ERK, and KIM-1 after treatment with calcium oxalate, which is consistent with a previous study [16]. Our results also suggested that CaSR might be involved in the induction of stone-related proteins in rats during ethylene glycol treatment since calcium oxalate-induced increases in the levels of OPN, ERK, and KIM-1 were further significantly increased by GdCl_3_ but were reversed by NPS-2390. OPN is not only an effective target molecule for the treatment of inflammation [36] but also regulates various steps of crystallization and contributes significantly to crystal deposition in the kidney [36]. Studies have shown that KIM-1 may be a useful marker for the early identification of kidney injury [37]. A study by Han et al. [38] studied the appearance of KIM-1 to identify renal injury developing postoperatively in the early period. This study showed that after injury for 3 h, urinary KIM-1 levels began to increase. The study also reported that KIM-1 is a beneficial marker for the early identification of AKI [38]. ERK mediates various cellular responses, including programmed cell death (apoptosis), epithelial sheet movement, and planar polarity [39,40], and cellular death may lead to more crystals adhering to the surface of renal cells and further stone formation.

It has been reported that OS caused by calcium oxalate is the initial trigger for the vicious cycle of nephrolithiasis [41,42]. In the present study, we found that OS was significantly increased after ethylene glycol treatment. These increased OS could be enhanced by the CaSR agonist GdCl_3_ or attenuated by the CaSR inhibitor NPS-2390, suggesting that calcium oxalate mediates OS though CaSR. CaSR promote crystal adherence, but it indirectly promote crystal adherence. The upregulation of the CaSR happened first, subsequently excessive amounts of crystals could trigger the production of ROS though CaSR in kidney tissues. ROS production leads to renal epithelial injury, which provide sites for crystal adherence and eventual deposition within the kidney.

For stone formation, OS could lead to renal injury and dysfunction. These renal pathological changes are accompanied by crystal retention. Crystal nucleation, aggregation, and retention are promoted by lethal epithelial cellular injury. The crystal nucleation, aggregation, and retention, in turn, further damages the kidneys. Dysfunctional cells and sublethal injury may generate ineffective crystallization inhibitors and localized areas of supersaturation in the interstitium. In our study, compared with group B, increased calcium oxalate-induced OS in group C may lead to more crystal retention and dysfunction. However, decreased OS in group D could inhibit this process.

In addition, we found that rats with nephrolithiasis showed low urinary calcium and citrate and high urinary oxalate compared to control animals. Oxalate is a more important promoter of kidney stones and exhibits 15-fold greater efficacy on urinary calcium oxalate saturation than calcium [43]. Notably, we observed low urinary oxalate in our rat nephrolithiasis model treated with GdCl_3_ compared with group B; one possible explanation for this finding is that CaSR increased free calcium, which binds to oxalate and thus reduces urinary oxalate. In addition, the main risk factor for recurrence of calcium oxalate stones is thought to be urinary citrate deficiency. Urinary citrate inhibits the formation of stones by inhibiting nucleation and growth [44]. Our results showed that urinary citrate levels decreased in the rat nephrolithiasis model. The CaSR inhibitor NPS-2390 increased the urinary citrate concentration and reduced crystal deposition in the rat nephrolithiasis model compared with group B. Therefore, we speculated that NPS-2390 might be a potential drug for the treatment of nephrolithiasis, especially when exposed to ethylene glycol, but the effect must be further verified by animal experiments and clinical trials before introducing it in clinical practice.

The findings of the present study may provide novel biomarkers for the diagnosis, prognosis, and clinical trials of calcium oxalate nephrolithiasis.

## Conclusions

The results suggest that CaSR might play significant roles in the induction of nephrolithiasis in rats through ROS production and regulation of PS ectropion, stone-related protein expression, and composition of urine in urine. These findings may serve as potential therapeutic and drug targets.

## Abbreviations

CaSR: calcium-sensing receptor
EG: ethylene glycol
OS: oxidative stress
PS: phosphatidylserine
SOD: superoxide dismutase
ROS: reactive oxygen species
MDA: malondialdehyde
MAPK: mitogen-activated protein kinase
ERK: extracellular signal-regulated protein kinase
JNK: c-Jun NH2-terminal protein kinase
OPN: osteopontin
KIM-1: kidney injury molecule 1
ESWL: extracorporeal shock wave lithotripsy
URS: ureteroscopy
PNL: percutaneous nephrolithotomy
BUN: blood urea nitrogen
Cr: serum creatinine
